# Binding of Organometallic Ruthenium Anticancer Complexes to DNA: Thermodynamic Base and Sequence Selectivity

**DOI:** 10.3390/ijms19072137

**Published:** 2018-07-23

**Authors:** Suyan Liu, Aihua Liang, Kui Wu, Wenjuan Zeng, Qun Luo, Fuyi Wang

**Affiliations:** 1 Institute of Chinese Materia Medica, China Academy of Chinese Medical Sciences, Beijing 100700, China; syliu@icmm.ac.cn (S.L.); ahliang@icmm.ac.cn (A.L.); 2 Beijing National Laboratory for Molecular Sciences, National Centre for Mass Spectrometry in Beijing, CAS Key Laboratory of Analytical Chemistry for Living Biosystems, CAS Research/Education Centre for Excellence in Molecular Sciences, Institute of Chemistry, Chinese Academy of Sciences, Beijing 100190, China; zengwj2014@iccas.ac.cn (W.Z.); qunluo@iccas.ac.cn (Q.L.); 3 School of Chemistry and Chemical Engineering, Wuhan University of Science and Technology, Wuhan 430081, China; 4 University of Chinese Academy of Sciences, Beijing 100049, China

**Keywords:** organometallic ruthenium complexes, anticancer, oligodeoxynucleotide, base/sequence selectivity, thermodynamics, LC-MS

## Abstract

Organometallic ruthenium(II) complexes [(η^6^-arene)Ru(en)Cl][PF_6_] (arene = benzene (**1**), *p*-cymene (**2**), indane (**3**), and biphenyl (**4**); en = ethylenediamine) are promising anticancer drug candidates both in vitro and in vivo. In this paper, the interactions between ruthenium(II) complexes and 15-mer single- and double-stranded oligodeoxynucleotides (ODNs) were thermodynamically investigated using high performance liquid chromatography (HPLC) and electrospray ionization mass spectroscopy (ESI-MS). All of the complexes bind preferentially to G_8_ on the single strand 5′-CTCTCTT_7_G_8_T_9_CTTCTC-3′ (**I**), with complex **4** containing the most hydrophobic ligand as the most reactive one. To the analogs of **I** (changing T_7_ and/or T_9_ to A and/or C), complex **4** shows a decreasing affinity to the G_8_ site in the following order: -AG_8_T- (*K*: 5.74 × 10^4^ M^−1^) > -CG_8_C- > -TG_8_A- > -AG_8_A- > -AG_8_C- > -TG_8_T- (**I**) ≈ -CG_8_A- (*K*: 2.81 × 10^4^ M^−1^). In the complementary strand of **I**, the G bases in the middle region are favored for ruthenation over guanine (G) bases in the end of oligodeoxynucleotides (ODNs). These results indicate that both the flanking bases (or base sequences) and the arene ligands play important roles in determining the binding preference, and the base- and sequence-selectivity, of ruthenium complex in binding to the ODNs.

## 1. Introduction

Organometallic ruthenium(II) arene complexes [(η^6^-arene)Ru(YZ)(X)]^n+^ have interesting anticancer properties both in vitro and in vivo, including cytotoxic activity towards cisplatin-resistant cell lines [1,2,3,4,5,6,7,8,9]. The arene ligand occupies three coordination sites in this type of pseudo-octahedral complexes and stabilizes Ru in its +2 oxidation state [10]. These mono-functional complexes appear to have a novel mechanism of action, differing from those of bi-functional cisplatin [11] and of the Ru(III) anticancer complexes, for example, (ImH)[*trans*-RuCl_4_Im(Me_2_SO)] (NAMI-A, Im = imidazole) and (IndH)[*trans*-RuCl_4_(Ind)_2_] (KP1019, Ind = indazole), which have entered into clinical trials [12,13,14,15]. The cytotoxicity of the chloro ethylenediamine complexes [(η^6^-arene)Ru(en)Cl][PF_6_] (arene = benzene, *p*-cymene, biphenyl, tetrahydroanthracene, etc.; en = ethylenediamine) increases with the size of coordinated arene. DNA has been shown to be a potential target for these Ru(II) arene complexes, most of which bind selectively to N7 of guanine [16,17,18,19]. The biphenyl Ru complex shows a decreasing activity towards mononucleotides in the following order: 5′-GMP(N7) > 5′-TMP(N3) >> 5′-CMP(N3) > 5′-AMP (N7/N1), being more discriminatory between guanine (G) and adenine (A) bases than cisplatin, which exhibits affinity to adenine second to guanine [11]. The formation of H-bonds between en-NH_2_ groups of ruthenium complexes and C6O of guanine was found to associate with this selective coordination [16,17]. The guanine bases in oligonucleotides of different sequences and in calf thymus DNA have been demonstrated to be the only sites ruthenated by the Ru(II) arene complexes [18,20,21,22,23]. Importantly, complexes containing π-rich arene ligands, like biphenyl or tetrahydroanthracene, showed combined coordination to guanine N7 and non-covalent interactions between the arene ligands and DNA bases, including arene intercalation and minor groove binding [16,17,20,21].

We have previously demonstrated that the thymine (T) bases in 15-mer single-stranded oligodeoxynucleotides (ODNs) can kinetically compete with G for binding to ruthenium arene complexes, but such T-bound mono-ruthenated ODN complexes were not thermodynamically stable and finally transformed to stable G-bound adducts, including mono-G-bound ODNs and minor G,T-bound di-ruthenated adducts [24]. Interestingly, the T-bases in G-quadruplex DNA are both kinetically and thermodynamically competitive with G-bases for ruthenation by ruthenium arene complexes because G-N7 is involved in H-bonding in G-quadruplex DNA [25].

The chloride ligand in the mono-functional compounds [(η^6^-arene)Ru(en)(Cl)]^+^ is readily hydrolyzed to give the more reactive aqua species [(η^6^-arene)Ru(en)(H_2_O)]^2+^. However, reaction of the aqua ruthenium complexes with DNA is retarded at high pH, suggesting that Ru–OH_2_ bonds are more reactive towards DNA than Ru–OH bonds [24,26,27]. Such behavior appears parallel to that of Pt(II) diam(m)ine anticancer complexes [28]. The bi-functional complex cisplatin undergoes a two-step aquation to form 1,2-intrastrand cross-linked DNA adducts, which is thought to be the main lesions on DNA that triggers apoptosis signaling initiated by high mobility group box 1 (HMGB1) protein recognition [11]. These two steps of hydrolysis, especially the second step, can be rate-limiting for interactions of cisplatin with DNA bases [28]. For instance, the formation of 5′-GpA intrastrand crosslinks by cisplatin is more preferred than that of 5′-ApG in d(ApGpA) and d(TpApGpApT) [29]. The rate of hydrolysis of the chloride ligand in the mono-aquated cisplatin was shown to be the key step that controlled the final bi-functional intrastrand adduct profiles when cisplatin bound to DNA with various X-purine-purine-Y motif [30]. However, aquation cannot be the only factor that controls the rate and selectivity of DNA binding of cisplatin. The diaquated form of cisplatin, [Pt(NH_3_)_2_(OH_2_)_2_]^2+^, displays no preference for the 3′- or 5′-guanine in a TpGpGpT sequence of a hairpin duplex oligonucleotide [31] or a single-stranded oligonucleotide [32]. Surprisingly, the 5′-guanine in TpGpGpC motif of a single-stranded oligonucleotide is preferred by a factor of two, and even by a factor of twelve over the 3′-guanine in a duplex oligonucleotide [33]. These indicate that DNA structure (or/and sequence) is a primary factor for determining the binding preference of bifunctional cisplatin to DNA. For the mono-functional Ru(II) arene complexes, binding to DNA may not be as complicated as that of cisplatin, but the varied flanking sequences next to the binding sites (e.g., guanine bases) may significantly affect the selective ruthenium binding, finally causing distinct response to the damaged DNA. However, the DNA binding properties, in particular sequence selectivity, of Ru(II) arene complexes are poorly understood.

In this present work, the interactions of [(η^6^-arene)Ru(en)Cl][PF_6_] (arene = benzene (**1**), *p*-cymene (**2**), indane (**3**), and biphenyl (**4**)) with 15-mer single- or double-stranded ODNs have been thermodynamically investigated using high performance liquid chromatography (HPLC) and LC-mass spectroscopy (MS). Based on the thermodynamic binding constants, we found the arene ligands of ruthenium complexes and adjacent bases of guanine have great influence on the binding affinity of ruthenium to guanine. We elucidated the base-selectivity and sequence-selectivity on the DNA binding of the ruthenium arene complexes, and hope to provide novel insights into the molecular mechanism of action of organometallic ruthenium anticancer complexes.

## 2. Results

### 2.1. Reactions of Organometallic Ruthenium(II) Complexes with One-G-centered Single-Stranded ODNs

**Chart 1:**
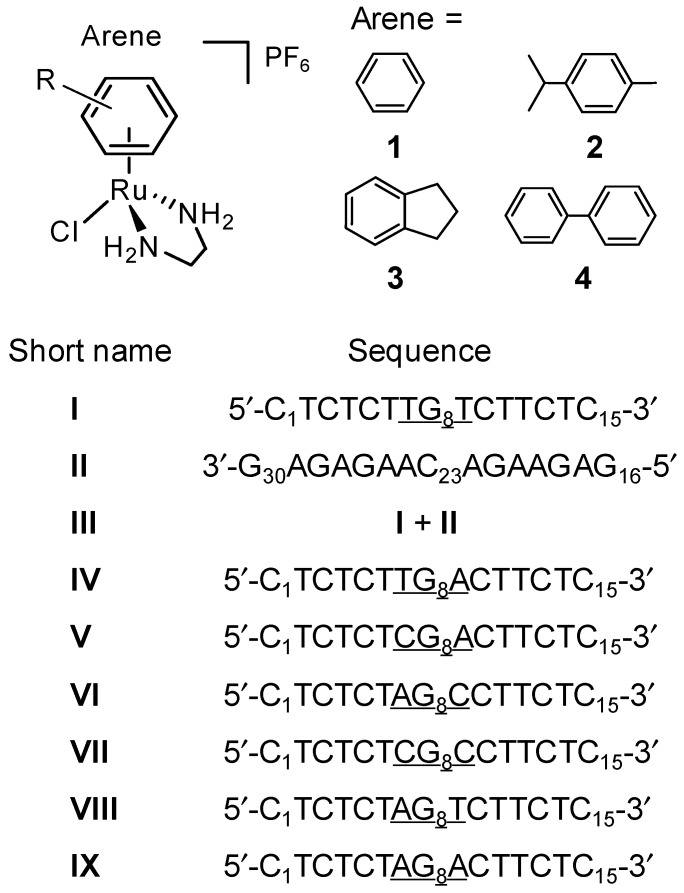
Structures of ruthenium arene anticancer complexes and sequences of oligodeoxynucleotides (ODNs) used in this work. ODNs **IV**–**IX** are the analogues of strand **I** with only variations at the neighboring bases of the central guanine (underlined).

**Figure 1:**
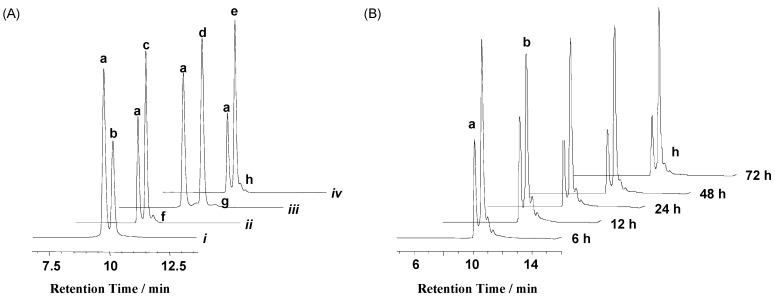
High performance liquid chromatography (HPLC) chromatograms with ultraviolet (UV) detection at 260 nm for reactions of ruthenium complexes with single strand **I**. (**A**) Complex **1** (*i*), **2** (*ii*), **3** (*iii*), or **4** (*iv*) with single strand **I** (0.1 mM, Ru/**I** = 1.0) in 50 mM triethylammonium acetate buffer (TEAA) (pH 7) at 310 K for 24 h; (**B**) complex **1** with **I** (0.1 mM, Ru/**I** = 1.0) in 50 mM TEAA (pH 7) and 5 mM NaCl at 310 K for different times. Peak assignments: a, unruthenated strand **I**; b, c, d, and e, mono-ruthenated **I**; f, g, and h, di-ruthenated **I**. For mass spectra, see Figure S1 in Supplementary Materials.

**Figure 2:**
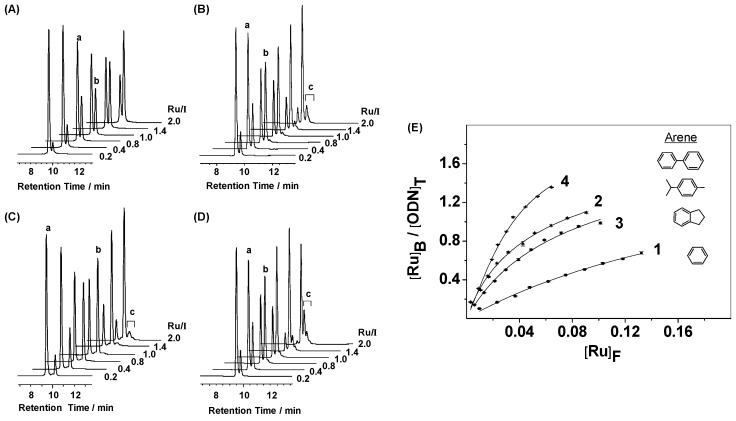
(**A**–**D**) HPLC chromatograms for reaction mixtures of complex **1** (**A**), **2** (**B**), **3** (**C**), or **4** (**D**) with single strand **I** (0.1 mM) at various molar ratios in 50 mM TEAA at 310 K for 24 h. Peak assignments: a, unruthenated strand **I**; b, mono-ruthenated **I**; c, di-ruthenated **I**. (**E**) A direct plot (dots) of moles of ODN-bound **1**, **2**, **3**, or **4** ([Ru]_B_) as a function of the concentration of free ruthenium complexes ([Ru]_F_). Computer-fitting (lines) of the experimental data to the ligand–receptor binding Equation (8) (see details in Supplementary Materials) gave rise to the equilibrium constants for the two-site binding reactions listed in Table 2.

Chart 1 shows the structures of the Ru(II) arene complexes and sequences of ODNs used in this work. Firstly, each ruthenium complex was incubated with 1 mol equiv strand **I** in 50 mM triethylammonium acetate buffer (TEAA) buffer solution (pH 7) at 310 K for 24 h. The reaction mixtures were then separated and analyzed by HPLC with ultraviolet (UV) detection at 260 nm (Figure 1), and each fraction was identified by electrospray ionization mass spectroscopy (ESI-MS). The observed negatively-charged ions corresponding to ruthenated **I** are listed in Table 1, and the mass spectra are shown in Figure S1 in the supplementary materials. The reaction of complex **1** with strand **I** gave rise to only one mono-ruthenated product, as indicated by the triply-charged ion [**I**-{(η^6^-benzene)Ru(en)}]^3−^ at *m*/*z* 1556.56 (Figure S1, supplementary materials). However, minor amount of di-ruthenated adducts were detected for the reaction mixtures of complexes **2**–**4** with 1 mol equiv strand **I** apart from the respective mono-ruthenated adducts at larger amount compared with the mono-ruthenated **I** by complex **1** (Figure 1, Figure S1 in supplementary materials). Moreover, even in the presence of 2-fold complex **1**, only ca. 67% of strand **I** was ruthenated (Figure 2) to produce mono-ruthenated ODN complex, while at the same molar ratio (Ru/**I** = 2.0), over 90% of strand **I** was ruthenated by complex **2**, **3**, or **4**, affording the mono-ruthenated ODN as the main product with a significant amount of di-ruthenated ODN complex (Figure 2).

In order to make sure that the reactions of complexes **1**–**4** with strand **I** reached equilibrium within 24 h, we analyzed the reaction mixtures of complex **4** with strand **I** for various times. As shown in Figure 1B, no pronounced changes in the product ratios were observed after 24 h of incubation even in the presence of 5 mM NaCl, which mimics the concentration of Cl^−^ in nuclear as Cl^−^ was thought to retard the hydrolysis of ruthenium arene complexes [26], subsequently slowing down their reaction with ODNs. These results indicate that the reactions of the ruthenium complexes with single-strand ODNs reached equilibrium within 24 h.

We have previously shown that the G_8_ base in strand **I** is the main binding site for the ruthenium biphenyl complex **4**, and that T_7_ and T_11_ in the same strand are the kinetically favored, yet thermodynamically unfavored, ruthenation sites by complex **4** [24]. Taking the structural similarity of the four ruthenium arene complexes into account, we assume that the mono-ruthenated adducts formed by the reactions of strand **I** with complexes **1**–**4** are G_8_-bound ODN complexes, and the minor di-ruthenated adducts are G_8_,T_x_-bound (x = 7 or 11) ruthenated **I** [24].

Next, the reaction mixtures of the four ruthenium complexes with strand **I** at various molar ratios of Ru to **I** were analyzed by HPLC so as to determine the equilibrium binding constants of the two-site receptor/ligand reaction. The results are shown in Figure 2A,D. On the basis of the HPLC peak areas with UV detection at 260 nm (the coordination of Ru(II) arene complexes to ODNs had little effect on their extinction coefficients at 260 nm), the extent of saturation (B in Equation (8) shown in Materials and Method) of the ODN binding sites was plotted as a function of the amount of unbound Ru complex (L) [25]. The resulting curves were computer-fitted to Equation (8) (Figure 2E), giving rise to the equilibrium constants listed in Table 2.

It can be seen that among the four ruthenium complexes, the benzene complex **1** had the lowest equilibrium constants for both binding steps to strand **I**, whereas the *p*-cymene complex **2** exhibits the highest binding affinity to strand **I** for the first step, and the biphenyl complex **4** is the most highly active to bind to strand **I** for the second step. It is notable that for complexes **2** and **3**, the *K* values for the first step binding are much higher than those for the second step, but complex **4** has a similar affinity for both of the binding steps. In other words, complexes **2** and **3** are more discriminative between the G and T in the single-stranded ODN than complex **4**.

**Scheme 1:**
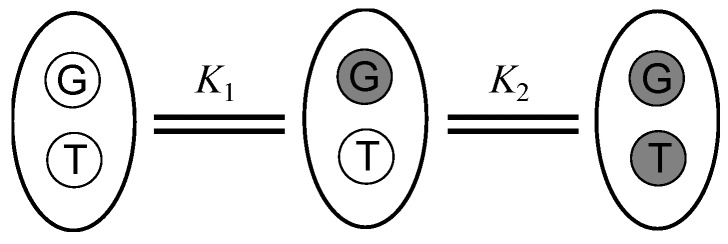
Schematic representative model for the binding reactions of organometallic ruthenium anticancer complexes to oligodeoxynucleotides (ODNs) **I** and **IV**–**IX** at the guanine (G) and thymine (T) sites based on the divalent receptor system. Bare circle with G/T means unoccupied site and black circle with G/T means occupied site.

**Figure 3:**
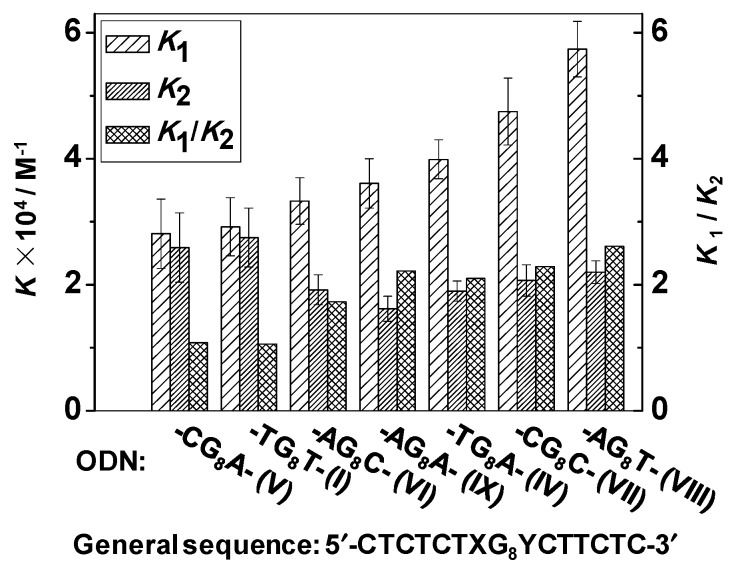
Sequence selectivity of organometallic ruthenium complex **4** binding to single-strand ODNs **I** and **IV**–**IX**. *K*_1_ and *K*_2_, of which the values are listed in Table S2 in supplementary materials, are the binding constants of complex **4** to each ODN at the first and the second step of binding, respectively.

To investigate the sequence selectivity of Ru(II) arene complexes binding to DNA, six analogues of single-stranded ODN **I** with sequence variants only at the adjacent bases to G_8_ (Chart 1) were selected to react with complex **4** at molar ratios of Ru/ODN ranging from 0.2 to 2 under the same conditions as described above. Analysis of the reaction mixtures by HPLC (Figure 2D, Figures S2A–S7A in supplementary materials) shows a similar binding profile for complex **4** to all the seven ODNs, that is, at Ru/ODN = 1.0, about 70% of the ODN was ruthenated, and at Ru/ODN = 2.0, less than 5% ODN remained intact. However, based on the thermodynamic G_8_,T_i_-diruthenated model (Scheme 1), the equilibrium constants (Table S1) resulting from the computer-fits of the titration data to Equation (8) showed a pronounced difference (Figure 3). The equilibrium constants for the first (mono-ruthenation) step of binding decreased in the following order: -AG_8_T- (VIII) > -CG_8_C- (VII) > -TG_8_A- (IV) > -AG_8_A- (IX) > -AG_8_C- > (VI) -TG_8_T- (I) ≈ -CG_8_A- (V), whereas the equilibrium constants for the second step of binding, which formed the di-ruthenated ODNs, decreased in the following order: -TG_8_T- (I) ≈ -CG_8_A- (IV) > -AG_8_T- (VIII) > -CG_8_C- (VII) > -AG_8_C- (VI) ≈ -TG_8_A- (IV) > -AG_8_A- (IX). Except for the -TG_8_T- and -CG_8_A- sequences, the equilibrium constants for the first step of binding of complex **4** to the other five ODNs are almost two-fold higher than those of the second step of binding, implying significant discrimination between the first ruthenation site (G_8_) and the second ruthenation site at T_x_ (Figure 3) [24].

### 2.2. Reactions of Organometallic Ruthenium(II) Complexes with Single-Strand II

**Figure 4:**
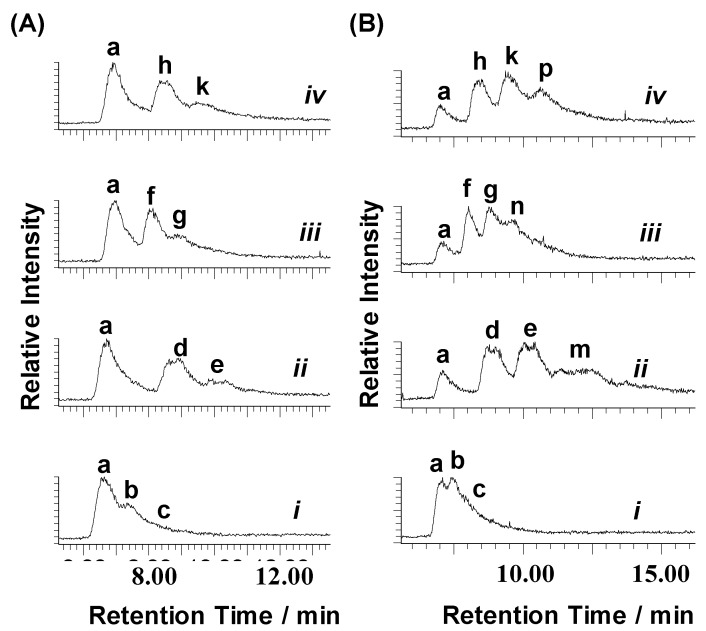
Total ion count (TIC) chromatograms for reactions of complex **1** (*i*), **2** (*ii*), **3** (*iii*), or **4** (*iv*) with single strand **II** (0.1 mM) at the molar ratio of (**A**) Ru/**II** = 1.0 or (**B**) Ru/**II** = 3.0 in 50 mM TEAA (pH 7) at 310 K for 24 h. Peak assignments: a, unruthenated strand **II**; b, d, f, and h, mono-ruthenated **II**; c, e, g, and k, di-ruthenated **II**; m, n, and p, tri-ruthenated **II**. Notably the fraction c in (**B**) contains a little tri-ruthenated **II** by **1** and fractions n and p contains a small amount of tetra-ruthenated **II** by **3** or **4** as shown in Figure S8 in supplementary materials.

The ruthenium complex **1**, **2**, **3**, or **4** was individually incubated with single-strand **II**, the complementary strand of **I**, at a molar ratio of Ru/**II** = 1.0 or 3.0 in 50 mM TEAA buffer solution at 310 K for 24 h. The reaction mixtures were then analyzed by HPLC and ESI-MS, and the observed negatively-charged ions for ruthenated **II** are listed in Table 3. The HPLC chromatograms with TIC (total ion count) detection and mass spectra of each HPLC fraction are shown in Figure 4 and Figure S8 in supplementary materials, respectively. At a molar ratio of Ru/**II** = 1.0, a significant amount of mono- and di-ruthenated **II** resulted from the reactions of **II** with complex **2**, **3**, or **4**, but the reaction of the benzene complex **1** with **II** afforded only mono-ruthenated **II**. Even in the presence of three-fold excess of complex **1**, nearly half of strand **II** remained intact after 24 h of reaction. In contrast, the reaction of strand **II** with three-fold excess of complex **2**, **3**, or **4** resulted in ruthenation of ca. 85% **II**, giving a pronounced amount of triply-ruthenated **II**. For **3** and **4**, a small amount of tetra-ruthenated **II**, as indicated by the triply-charged ions at *m*/*z* 1938.42 and 1986.32, respectively, were also observed (Table 3, Figure S8).

To identify the preferential binding sites of the tested ruthenium complexes in strand **II**, a three-fold excess of complex **1** or **2** was incubated with strand **II** at 310 K for 24 h. The adducts were digested by snake venom phosphodiesterase (SVP) after removing unbound ruthenium through centrifugation, and then analyzed by HPLC-ESI-MS in negative mode. The observed ions corresponding to ruthenated ODN fragments are listed in Table S2 in supplementary materials, and the mass spectra are shown in Figure S9 in the supplementary materials. Firstly, the results showed that the SVP digestion stopped at G_21_, as evidenced by the detection of doubly-charged ions at *m/z* 1050.53 and 1078.24, which are assignable to the mono-ruthenated ODN fragments [F_21_-**1**′]^2−^ and [F_21_-**2**′}]^2−^ (F_21_ = 3′-G_21_A_20_A_19_G_18_A_17_G_16_-5′, **1**′ = {(η^6^-ben)Ru(en)}, **2**′ = {(η^6^-*p*-cym)Ru(en)), respectively. These are parallel to the reactions of ODN **II** with complexes **3**–**4** [19]. Secondly, doubly-charged ions at *m*/*z* 1225.29 assignable to the di-ruthenated ODN fragment [F_21_-**2**′_2_]^2−^ were observed, indicating that the fragment 3′-G_21_A_20_A_19_G_18_A_17_G_16_-5′ contains two binding sites for complex **2**, most likely G_21_ and G_18_ as for complex **3** reported previously [19]. Thirdly, the triply-charged ions at *m/z* 1335.63, which correspond to the ruthenated fragments [F_26_-**2**′_2_]^3−^ (F_26_ = 3′-G_26_A_25_A_24_C_23_A_22_G_21_A_20_A_19_G_18_A_17_G_16_-5′), were detected, providing evidence for the formation of di- ruthenated **II** at G_21_ and G_26_ by complex **2** as observed for the reaction of the same strand with complex **4** [19]. These results suggest that G_21_ in the middle region of single strand **II** is the common preferential binding site for complexes **1**–**4**. Additionally, G_18_ and G_26_ are the secondary binding sites for complexes **3** and **4**, respectively, and both G_21_ and G_26_ are the secondary binding sites for complex **2**.

### 2.3. Reactions of Organometallic Ruthenium(II) Complexes with Duplex III

**Figure 5:**
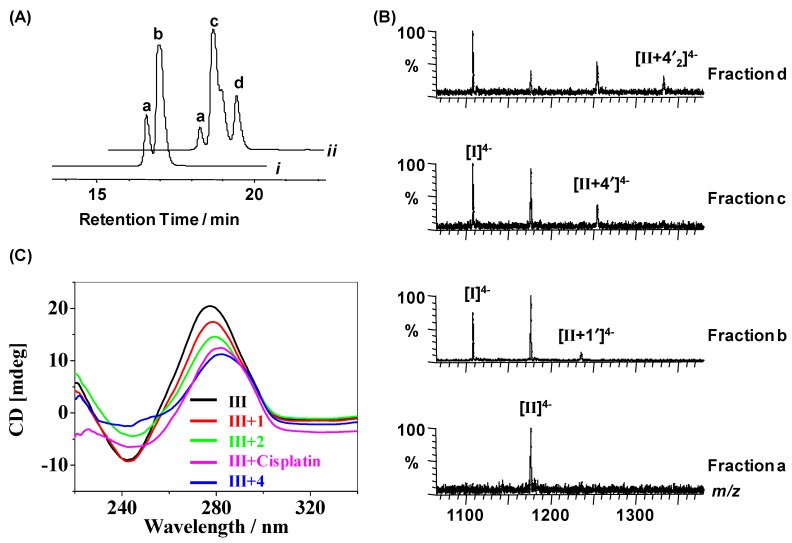
(**A**) HPLC chromatograms with ultraviolet (UV) detection at 260 nm for the reaction mixture of duplex **III** with complex **1** (*i*) or complex **4** (*ii*) (Ru/**III** = 1.0) in 50 mM TEAA buffer (pH 7) and 100 mM NaClO_4_ incubated at 310 K for 48 h; (**B**) mass spectra for HPLC fractions shown in (**A**); (**C**) circular dichroism (CD) spectra of free duplex **III** (black) and the reaction mixtures of duplex **III** with complex **1** (red), **2** (green), **4** (blue) and cisplatin (magenta) incubated at 310 K for 24 h at a molar ratio of Ru/**III** or Pt/**III** = 6.0.

Next, mixtures of complex **1** or **4** with duplex **III** (= **I** + **II**) at Ru/**III** = 1.0 or 6.0 in 50 mM TEAA buffer (pH 7) containing 100 mM NaClO_4_ were incubated at 310 K for 48 h, and then analyzed by HPLC followed by ESI-MS analysis under negative-ion mode. The chromatograms for the reaction mixture of duplex **III** with complex **1** or **4** are shown in Figure 5A, and Figures S10A and S11A, and the corresponding mass spectra for HPLC fractions are shown in Figure 5B, and Figures S10B and S11B, respectively. The observed ions for the reaction mixtures at the molar ratio of Ru/**III** = 6.0 are listed in Table 4. It can be seen that at a low reaction molar ratio (Ru/**III** = 1.0) (Figure 5B), no ruthenated **I** by complex **1** or **4** was detected, but a little amount of mono-ruthenated **II** by complex **1** ([**II**-**1**′]^4−^) and both mono- and di-ruthenated **II** adducts [**II**-**4**′]^4−^ and [**II**-**4**′_2_]^4−^ by complex **4** were obviously identified (Figure 5B). Even in the presence of six-fold excess of complex **1**, only a small amount of strand **I** was ruthenated, accompanied with the observation of a pronounced amount of mono-ruthenated **II** and a minor amount of di-ruthenated **II** adducts (Figure S10B). In contrast, for the binding of complex **4** to duplex **III** at **4**/**III** = 6.0, a series of multi-ruthenated **I** and **II** adducts were detected by MS following HPLC separation. The last HPLC fraction (peak e in Figure S11A) was identified as a mixture of tri-ruthenated **I**, and tetra-, penta-, and hexa-ruthenated **II** by **4** (Figure S11B). Meanwhile, little free **II** (peak a, Figure S11A) remained, while a large amount of unreacted strand **I** (peak b, Figure S11A) was detected in the reaction mixture of complex **4** to duplex **III** at **4**/**III** = 6.0. It is noticed that under the given chromatographic conditions, the HPLC separation denatured the duplex, as well as its ruthenated adducts, into single-stranded ODNs, but a small amount of ruthenated duplex **III** was still detected in the fraction c and d in the forms of di-, tri-, tetra-, and penta-ruthenated duplex **III** (Figure S11B in supplementary materials).

Circular dichroism (CD) spectroscopy was introduced to elucidate the effect of the Ru arene complexes on the conformation of duplex ODN **III**. A CD spectrum provides unique signals in diagnosing changes in DNA conformation during drug-DNA interactions, for example, the maximum positive adsorption at ~275 nm attributed to DNA base stacking, and the maximum negative adsorption at ~245 nm assigned to the right-handed helicity of duplex DNA, may change in both intensity and wavelength subject to reactions of DNA with drug molecules [34]. CD spectra for 15-mer duplex **III** (in 50 mM TEAA buffer containing 100 mM NaClO_4_) in the absence and presence of increasing amounts of Ru arene complexes **1**, **2**, or **4** are compared as shown in Figure 5C and Figure S12 in supplementary materials, with the applied molar ratios of Ru/**III** = 1.0, 3.0, and 6.0, respectively. Cisplatin was also introduced to interact with the duplex DNA **III** as a reference. The 15-mer duplex used in our experiments has a typical B-DNA conformation in the 100 mM NaClO_4_ solution with the maximum positive absorption (base stacking) at ~277 nm and the maximum negative absorption (right-handed helicity) at ~242 nm. The increasing concentration of the ruthenium complexes made more Ru bind to the duplex DNA, leading to an obvious decrease in the intensity of both positive and negative CD signals. Complex **4** resulted in the largest decrease in the signal intensity and caused the two absorption bands to both approach the baseline gradually (Figure S12C). Another interesting variation was that the maximal positive absorption wavelength had a little red-shift from 277 nm to 282 nm because of the increasing level of ruthenation by complex **4**, while the other two Ru complexes caused no obvious band shift in the CD spectrum of the duplex ODN. At the same molar ratio (Ru/**III** = Pt/**III** = 6.0), the intensity decrease of positive signal caused by the ruthenium complexes and cisplatin was in the following order: **1** < **2** < cisplatin < **4**, while for negative signal, the order was as follows: **1** < cisplatin < **2** < **4** (Figure 5C).

## 3. Discussion

Through recognizing different sequences of nucleic acids, the repressor and activator proteins can regulate the gene expressions. The selective manipulation of gene expression can be achieved by using small molecules that can target DNA to selectively activate or repress gene expression have a high potential for being anticancer therapeutics [35,36,37,38,39]. Therefore, it is of great importance to study the base and sequence selectivity of anticancer metallodrugs and candidates as DNA binders.

The most studied organometallic ruthenium(II) complexes are [(η^6^-arene)Ru(en)(Cl)]^+^, where arene = biphenyl (bip), tetrahydroanthracene (tha), dihydroanthracene (dha), *p*-cymene (cym), or benzene (ben) have been previously reported to bind preferentially to N7 of guanosine and to N3 of thymidine, but weakly to N3 of cytidine, and little to adenosine . Such base-selectivity appears to be enhanced by strong intra-molecular H-bonding between the en NH_2_ groups and exocyclic oxygens. However, reacting with the single-stranded ODNs d(ATACATG_7_G_8_TACATA) (**X**), d(TATG_25_TACCATG_18_TAT) (**XI**) or the duplex **XII** (= **X** + **XI**), the biphenyl Ru(II) complex was demonstrated to bind only to N7 of guanine bases (G_7_, G_8_, G_18_, and G_25_), but not to thymine residues in either of the single- or double-stranded ODNs [20]. We have recently shown that thymine bases in single-strand **I** and its analogs are kinetically competitive with guanine bases for binding to organometallic ruthenium(II) complexes [24]. The T-bound mono-ruthenated adducts are not stable and could dissociate or transfer to G-bound adducts or G_8_,T_x_-diruthenated adducts [25]. In the present work, our thermodynamic studies further prove that the di-ruthenated adducts formed by the reactions of complexes **2**–**4** with single strand **I** and **IV**–**IX** bearing a single G base were still detectable even after 24 h of reaction at 310 K. The binding constants of Ru complexes **1**–**4** to both G_8_ and T_x_ in the single-stranded ODNs increase with the size of the arene ligands, consistent with previous reports [16]. The similar binding preference of Ru to G over T was also observed for the reactions of the ruthenium complexes with the duplex ODN **III**. In these cases, the thymine ruthenation occurred only when the most of guanine bases in the duplex **III** were ruthenated. This is just the opposite with the base selectivity of Zn(II)–acridinylcyclen complexes, which bound to G only after most of T was occupied in GpT, d(GTGTCGCC), or in duplex d(CGCTAGCG)_2_ in neutral or basic solution [40]. However, in acidic solution or nonpolar solvent, the attack of Zn to T was restricted because of the protonation of T-N3 [41]. We have also found that the competition resulting from the protonation of T-N3 reduces the binding affinity of the ruthenium arene complexes to T-N3 in single-stranded ODNs [24]. At pH 4.8, no T-bound adducts were detected in the reaction mixtures of the biphenyl complex **4** with strand **I** at Ru/**I** = 1.0.

The intercalation between the aromatic ligands of Ru arene complexes and the purine ring of DNA was one of driving forces for the (arene)Ru-G-N7/T-N_3_ coordination, especially for these with arene ligands with more than one ring, like biphenyl and tetrahydroanthracene [16,20,23,42,43,44,45]. The intercalating ligands in coordinately saturated octahedral Ru^II^, Os^II^, and Rh^III^ complexes showed similar function in increasing their DNA-binding affinity, and conferred them with capacity recognizing DNA bases via shape selection [46]. The strong π–π stacking of acridine–thymine rings was also found to enhance the affinity of Zn(II)–acridinylcyclen complexes to N3 of dT [47]. However, for some metal complexes, e.g., *trans*-[Pt(quinoline)(NH_3_)-(9EtG)Cl]^+^, the rigid quinoline ring retards the binding of Pt to N7 of 5′-GMP [48]. On the other hand, when the extended arene ligand rings in transition metal complexes intercalate into DNA bases, it may extend the phosphate spacing along the helix axis; as a consequence, lengthening and unwinding DNA helices [49,50]. Such intercalation distorts the structure of DNA helices, and makes the hidden sites, for example, T-N3, which participated in H-bonding with adenine base in duplex DNA, exposed for metal attacking. The reaction of complex **4** with duplex **III** produced pronounced amounts of G, T-bound di-/tri- ruthenated strand **I**, but only G-bound mono-ruthenated strand **I** formed by reaction of complex **1** with the same duplex ODN, strongly supporting this deduction [16,51].

Although both cisplatin and the Ru arene complexes can bind to DNA, and unwind and loosen the DNA helices, the changes in the helical structure of the bound DNA are different. For example, cisplatin unwinds DNA duplex to about 25° [52], while ruthenium complexes only unwind at most 14° [23]. The main reason is that cisplatin mainly forms intrastrand crosslinks, which bend the helices toward the platinated sites, while ruthenium complexes form mono-functional adducts accompanied by the intercalation of arene ligands between nucelobases, bending DNA duplexes toward the direction opposite to the ruthenated site. Therefore, the changes in the helicity of DNA due to Ru binding are dependent on the size of arene ligands of the Ru complexes as evidenced by CD spectra shown in Figure 5C.

Furthermore, our thermodynamic data demonstrate that the arene ligands of the ruthenium complexes regulate not only base selectivity, but also sequence selectivity of these complexes to DNA. For instance, there are six G residues in the single strand **II**. Our studies show that G_21_ in the middle region is the common binding site for all the four ruthenium complexes, and that G_18_ in the 5’-region and G_26_ in the 3′-region are the secondary binding site for complexes **3** and **4**, respectively, while both G_18_ and G_26_ are the secondary binding site for complex **2**. These results imply that the ruthenium arene complexes preferentially bind to guanine bases in the middle region of the ODN strand.

For the reactions of polyanionic DNA and the cationic [(η^6^-arene)Ru(en)(H_2_O)]^2+^, which is the reactive species of the Ru arene complexes produced by hydrolysis in aqueous solution, electrostatic interactions and H-bonding are the initial recognition forces between Ru complexes and DNA prior to Ru–DNA coordination [16]. Such binding is noncovalent and reversible. It may significantly influence the site preference of DNA-metal bindings, which are kinetically controlled [53]. It is proposed that the preferential binding of cisplatin to guanines is predominantly a result of electrostatic attraction of the platinum toward the most nucleophilic sites [54,55], for example, the nitrogen N7 atoms of purines as they are the most electron-dense and accessible sites in DNA for electrophilic attack by platinum, or by ruthenium in this work. Moreover, the G-N7 sites are exposed in the major groove of the double helix, and are not involved in base-pair hydrogen-bonding [56,57], which makes G-N7 sites more favored for metalation. However, variations at the neighboring bases of G may cause a change in the microenvironment that in turn modulates metal coordination and alters the affinity of Ru atom to guanine. Indeed, our thermodynamic studies show that the binding constants of complex **4** to the centered G base in seven 15-mer single-stranded ODNs containing a variation in the flanking bases of the G site are significantly different. The binding constant of complex **4** to G in -A_7_G_8_T_9_- sequence is nearly two-fold higher than that to G in -C_7_G_8_A_9_- sequence. An interesting result was that for the single-G sequences with adenine being the flanking base in one side, the affinity of Ru to G decreased with the variation of the other side base in an order as follows: T > A > C, whether A was at the 3′- or 5′-side of the central G site. This is in line with the reactivity order of the biphenyl organometallic ruthenium complex to mononucleotides where Ru binding to A and C is much weaker than binding to G and T, implying that the binding of the ruthenium complex to an adjacent T base may be synergetic to the thermodynamically favored G-binding [24,25]. It has been previously reported that the flanking bases play an important role in the sequence selectivity of the bindings of *cis*-platinum anticancer drugs to DNA [58]. One of the early studies on this topic demonstrated that the reaction of cisplatin (*cis*-[PtCl_2_(NH_3_)_2_]) with salmon sperm DNA gave rise to chelating adducts with Pt–GG (65%) and Pt–AG (25%), but no Pt–GA adducts (0%) additional to Pt–G mono-functional adduct (10%) [59]. However, the mechanism of crosslinking did not necessarily follow the sequence selectivity of the initial coordination. The fact that the 5′-monoplatinated adducts are formed more rapidly than the 3′-monoplatinated adducts may reflect the inherently greater reactivity of the 5′-G compared with the 3′-G [60]. To explain the differences, Kozelka and Chottard et al. proposed a model according to which the neighboring bases have an influence on the sequence selectively of Pt–G coordination by changing the electronegativity of the N7 site, and presenting different steric hindrances when platinum complex approaches the G-N7 site [61]. Other non-metallic DNA-binding drugs, like aflatoxin [62] and nitrogen mustards [63], also showed discriminated reactivity to G-N7 upon the local sequence context.

## 4. Materials and Methods

### 4.1. Materials

[(η^6^-arene)Ru(en)Cl][PF_6_] (arene = benzene (ben) (**1**), *p*-cymene (cym) (**2**), indane (ind) (**3**), and biphenyl (bip) (**4**); en = ethylenediamine; Chart 1) were synthesized as described in the literature [2,17,64,65]. Triethylammonium acetate buffer (TEAA, 1 M) was purchased from AppliChem (Darmstadt, Germany), and acetonitrile (HPLC grade) from Tedia (Fairfield, OH, USA). HPLC-purified 15-mer oligodeoxynucleotides (ODNs, Chart 1) were obtained from TaKaRa (Dalian, China), and the concentration was determined by UV at 260 nm absorption according to the instruction of manufacturer. Snake venom phosphodiesterase (SVP) was purchased from Orientoxin (Shandong, China). Microcon YM-3 ultrafilters were purchased from Millipore (Billerica, MA, USA). Aqueous solutions were prepared using MilliQ water (MilliQ Reagent Water System, Molsheim, France).

### 4.2. Sample Preparation

Stock solutions of Ru(II) arene complexes (5 mM) were prepared by dissolving individual complex in deionized water and diluted as required before use. The ODNs were dissolved in water to give 2 mM stock solutions. The reaction mixtures containing 0.1 mM single-stranded ODNs in 50 mM TEAA (pH 7) or 0.4 mM duplex **III** in 50 mM TEAA buffer containing 100 mM NaClO_4_ (pH 7) and various concentrations of Ru complexes were incubated at 310 K in the dark for 24 h, unless otherwise stated, and then separated or analyzed by HPLC or/and HPLC-ESI-MS. The general procedure to identify the binding sites of Ru complexes on ODNs was similar to that reported in our previous works by using exonuclease digestions combined with HPLC-ESI-MS [19] and the top-down MS method [24,66].

### 4.3. High Performance Liquid Chromatography (HPLC)

An Agilent 1200 series HPLC system was applied coupled with a quaternary pump, a 20-μL Rheodyne sample injector, and a UV-Vis diode-array-detector (DAD) detector. All the HPLC data was processed by the Chemstation data processing system. Water containing 20 mM TEAA and acetonitrile containing 20 mM TEAA were used as mobile phases A and B, respectively. HPLC assays for reaction mixtures of Ru(II) arene complexes with different single-stranded ODNs were carried out on a Varian Pursuit XRs C18 reversed-phase column (100 × 2.0 mm, 3 μm, 0.2 mL·min^−1^, Varian, Inc., Palo Alto, CA, USA). The gradient for separating reaction mixtures of complexes **2** and **4** with strand **I** (Chart 1) was as follows: 8% solvent B from 0 to 3 min, increasing to 60% at 10 min, 80% from 11 min to 15 min, and resetting to 8% at 16 min. The same gradient was also applied to separate the reaction mixtures of complex **4** with other six single-stranded ODNs. For reaction mixtures of complexes **1** and **3** with strand **I**, the applied gradient was as follows: from 8% to 18% during first 10 min, 80% from 11 min to 15 min, and resetting to 8% at 16 min. The gradient was also applied to separate the reaction mixtures of complexes **1** and **4** with strand **II** (Chart 1). The same column was used to separate the products from enzymatic digestions of ruthenated ODNs by exonucleases prior to MS analysis with a gradient as follows: 1% to 5% solvent B from 0 to 5 min, 5% from 5 to 8 min, then increasing to 80% and keeping it from 15 to 21 min, and finally resetting to 1% at 22 min. An Agela C8 column (50 × 2.1 mm, 0.2 mL min^−1^, Agela Technology, Tianjin, China) was used to separate the reaction mixtures of ruthenium complexes with duplex **III** (Chart 1) prior to MS analysis with a gradient as follows: 1% solvent B for 3 min, 5% to 10% from 4 to 9 min and kept at 10% for 5 min, then increased to 80% at 15 min and retained for 4 min, and finally reset to 1% at 20 min.

### 4.4. Electrospray Ionization Mass Spectroscopy (ESI-MS)

A Micromass Q-TOF (Waters Corp., Manchester, UK) coupled with an Agilent 1200 system was applied under negative-ion mode for the online HPLC-ESI-MS assays. The HPLC conditions were the same as described above, with a flow rate of 0.2 mL·min^−1^ and a splitting ratio of 1/3 into mass spectrometer. All the MS data was analyzed and post-processed by a Masslynx (ver. 4.0, Wasters Corp., Manchester, UK) data processing system. The MS conditions were similar as in our previously reported paper [19] and the typical conditions are as follows: spray voltage 2.8~3.8 kV; cone voltage 55~70 V; desolvation temperature 393 K; source temperature 373 K; cone gas flow rate 50 L·h^−1^; desolvation gas flow rate 500 L·h^−1^; and collision energy 10 V. Mass spectra were acquired in the range of 200–3000 *m*/*z* with mass accuracy of all measurements within 0.01 *m*/*z* unit, which were calibrated versus a NaI calibration file. All the *m*/*z* values are the mass-to-charge ratios of the most abundant isotopomer for observed ions.

### 4.5. Circular Dichroism (CD) Spectroscopy

For CD spectroscopy analysis, the reaction mixtures, containing 15 μL 0.1 mM duplex **III** in 100 mM NaClO_4_ and 50 mM TEAA (pH 7), and 15 μL various concentrations of Ru complexes in 50 mM TEAA, were incubated at 310 K for 24 h in dark, and then diluted to 5 μM using 50 mM TEAA buffer prior to CD analysis. CD spectra were recorded by using a Jasco J-810 spectropolarimeter in the range 220–340 nm in 0.5 nm increments with an average time of 1 s, the cell path-length was 1 cm. Each spectrum was the average of ten scans and corrected by the blank buffer solution.

### 4.6. Determination of Equilibrium Binding Constants of Organometallic Ruthenium Complexes to ODNs

The peak areas of ruthenated ODNs separated by HPLC with UV detection at 260 nm were used to determine the equilibrium binding constants of Ru(II) arene complexes to single-stranded ODNs. Klotz’s methods (affinities from a stoichiometric perspective) were applied to construct the reaction model and determine the stoichiometric equilibrium constants (Scheme 1) [67]. The G- and T-bound two-site divalent model was applied as there are only G- and T-binding sites of ruthenium complexes on each single strand ODN identified as described previously [24]. Although there are more than one T binding sites on single strands **I** and **IV**–**VI**, it is difficult to fully separate the different G_8_, T_i_-bound ODN adducts by HPLC [24]. Therefore, for clarity, the HPLC peak areas corresponding to di-ruthenated ODN adducts with different T-sites were summed to calculate the content of di-ruthenated species.

For the divalent receptor system, the uptake of ligand L (Ru complexes) by receptor R (ODNs) can be described by two steps:

R + L = RL_1_ (1)

RL_1_ + L = RL_2_ (2)

with the following equilibrium constants:

*K*_1_ = [RL_1_]/[R] [L] (3)

*K*_2_ = [RL_2_]/[RL_1_] [L] (4)

Thus,

[RL_1_] = *K*_1_ [R] [L] (5)

[RL_2_] = *K*_2_ [RL_1_] [L] = *K*_1_*K*_2_ [R] [L]^2^ (6)

Therefore, the extent of site occupation (B) can be defined as follows:

B = mole of bound ligand/mole of total receptor = ([RL_1_] + 2[RL_2_])/([R] + [RL_1_] + [RL_2_]) (7)

Replacing [RL_1_] and [RL_2_] by Equations (5) and (6) into Equation (7), we obtain the stoichiometric binding equation:

B = (*K*_1_ [L] + 2 *K*_1_*K*_2_ [L] ^2^)/(1 + *K*_1_ [L] + *K*_1_*K*_2_ [L] ^2^) (8)

where *K*_1_ and *K*_2_ are the binding constants for the first and the second step of reaction, respectively. The B −−− [L] data obtained by HPLC analysis were fitted computationally using Equation (8) to give rise to the stoichiometric equilibrium constants *K*_1_ and *K*_2_.

## 5. Conclusions

In summary, we have thermodynamically studied the reactions of the organometallic ruthenium(II) anticancer complexes [(η^6^-arene)Ru(en)Cl][PF_6_] (arene = benzene (**1**), *p*-cymene (**2**), indane (**3**), biphenyl (**4**); en = ethylenediamine) with the 15-mer one-G-containing single-stranded ODN **I**; its complementary strand **II** and the duplex **III** (= **I** + **II**); and a series of analogue strands of **I** by HPLC, ESI-MS, and CD spectroscopy. Different arene ligands regulate the reactivity of the ruthenium complexes towards G and T of strand **I**, with complex **2** being the most discriminatory one between G and T bases, and complex **4** the most reactive one. In the complementary single-strand **II**, G_21_ is the common preferential binding site for complexes **1**–**4**; G_18_ and G_26_ are the secondary binding sites for complexes **3** and **4**, respectively; and both G_21_ and G_26_ are the secondary binding sites for complex **2**. This implies that the G bases in the middle region are more favored for ruthenation than G bases in the end of ODNs. When reacted with different single-G-containing ODNs, complex **4** shows a decreasing affinity to the G_8_ site in the following order: -AG_8_T- (*K*: 5.74 × 10^4^ M^−1^) > -CG_8_C- > -TG_8_A- > -AG_8_A- > -AG_8_C- > -TG_8_T- (**I**) ≈ -CG_8_A- (*K*: 2.81 × 10^4^ M^−1^), indicating that adjacent bases play an important role in the sequence selectivity of the bindings of the organometallic ruthenium(II) complexes to DNA, perhaps related to the arene intercalation and the steric hindrance of adjacent bases. Among the tested ruthenium complexes, complex **4** caused the most severe distortion to DNA duplex conformation as a result of the intercalation of biphenyl ligand into bases. These findings provide a molecular basis for better understanding in the base and sequence selectivity of the binding of ruthenium arene anticancer complexes to DNA, and is helpful to design and development of novel ruthenium-based anticancer complexes.

## Figures and Tables

**Table 1:** Negatively-charged ions observed by electrospray ionization mass spectroscopy (ESI-MS) for the high performance liquid chromatography (HPLC) fractions (Figure 1) from the reaction mixtures of complexes **1**–**4** with 1 mol equiv of single-strand **I**. For mass spectra, see Figure S1.

Ru Complex	*m*/*z*: Observed (Calculated)		
	[I]^3−^	[I + {Ru} ^1^]^3−^	[I + {Ru}_2_]^3−^
**1**	a: 1477.22 (1477.24)	b: 1556.56 (1556.58)	— ^2^
**2**	a: 1477.22 (1477.24)	c: 1575.25 (1575.27)	f: 1672.96 (1672.95)
**3**	a: 1477.22 (1477.24)	d: 1569.91 (1569.92)	g: 1662.65 (1662.60)
**4**	a: 1477.22 (1477.24)	e: 1581.89 (1581.92)	h: 1686.66 (1686.60)

^1^ {Ru} = {(η^6^-arene)Ru(en)}^2+^, arene = benzene (**1**), *p*-cymene (**2**), indane (**3**), or biphenyl (**4**); ^2^ not detectable.

**Table 2:** Equilibrium binding constants (*K*) for the reactions of complexes **1**–**4** with strand **I**.

Ru Complex	*K*_1_ (10^4^ M^−1^)	*K*_2_ (10^4^ M^−1^)	*K*_1_/*K*_2_
**1**	0.79 ± 0.04	0.18 ± 0.03	4.39
**2**	3.94 ± 0.24	0.51 ± 0.05	7.72
**3**	2.52 ± 0.13	0.43 ± 0.04	5.86
**4**	2.92 ± 0.46	2.75 ± 0.47	1.06

**Table 3:** Negative ions observed by ESI-MS for the HPLC fractions shown in Figure 4 (for mass spectra, see Figure S8 in supplementary materials).

Ru Complex	*m*/*z*: Observed (Calculated)			
	[II + {Ru} ^1^]^3−^	[II + {Ru}_2_]^3−^	[II + {Ru}_3_]^3−^	[II + {Ru}_4_]^3−^
**1**	b: 1647.31 (1647.28)	c: 1726.69 (1726.62)	c: 1805.81 (1805.62)	— ^2^
**2**	d: 1666.32 (1666.30)	e: 1764.34 (1764.33)	m: 1862.10 (1862.02)	— ^2^
**3**	f: 1660.96 (1660.96)	g: 1753.37 (1753.30)	n: 1846.01 (1845.99)	n: 1938.42 (1938.34)
**4**	h: 1672.96 (1672.96)	k: 1777.33 (1777.30)	p: 1882.04 (1881.99)	p: 1986.32 (1986.33)

^1^ {Ru} = {(η^6^-arene)Ru(en)}^2+^ (arene = benzene (**1**), *p*-cymene (**2**), indane (**3**) or biphenyl (**4**); ^2^ Not detected.

**Table 4:** Negatively-charged ions observed by ESI-MS coupled to denatured HPLC for reaction mixture of Ru complex **1** or **4** with duplex **III** (Ru/**III** = 6.0, **III** = **I** + **II**). For chromatograms and mass spectra, see Figures S10 and S11 in supplementary materials.

Ru Complex	Observed (Calculated) *m*/*z*	Observed Ions
**1**	1107.89 (1107.91)	[**I**]^4−^
	1167.44 (1167.43)	[**I**-**1**′ ^1^]^4−^
	1175.92 (1175.96)	[**II**]^4−^
	1235.44 (1235.46)	[**II**-**1**′]^4−^
	1294.69 (1294.71)	[**II**-**1**′_2_]^4−^
	1342.93 (1342.96)	[**II**-**1**′_3_]^4−^
**4**	1186.40 (1186.44)	[**I**-**4**′^1^]^4−^
	1264.67 (1264.70)	[**I**-**4**′_2_]^4−^
	1343.18 (1343.21)	[**I**-**4**′_3_]^4−^
	1477.57 (1477.57)	[**I**]^3−^
	1582.23 (1582.25)	[**I**-**4**′]^3−^
	1686.69 (1686.60)	[**I**-**4**′_2_]^3−^
	1791.38 (1791.28)	[**I**-**4**′_3_]^3−^
	1254.19 (1254.22)	[**II**-**4**′]^4−^
	1332.76 (1332.73)	[**II**-**4**′_2_]^4−^
	1411.23 (1411.24)	[**II**-**4**′_3_]^4−^
	1489.46 (1489.50)	[**II**-**4**′_4_]^4−^
	1567.73 (1567.76)	[**II**-**4**′_5_]^4−^
	1646.30 (1646.27)	[**II**-**4**′_6_]^4−^
	1568.36 (1568.28)	[**II**]^3−^
	1672.66 (1672.63)	[**II**-**4**′]^3−^
	1777.35 (1777.30)	[**II**-**4**′_2_]^3−^
	1882.07(1881.99)	[**II**-**4**′_3_]^3−^
	1986.45(1986.33)	[**II**-**4**′_4_]^3−^
	2090.80(2090.68)	[**II**-**4**′_5_]^3−^

^1^**1**′ = {(η^6^-benzene)Ru(en)}^2+^; **4**′ = {(η^6^-biphenyl)Ru(en)}^2+^.

## Supplementary Materials

Supplementary materials can be found at http://www.mdpi.com/1422-0067/19/7/2137/s1.
