# Supplementary material for: Binding of Organometallic Ruthenium Anticancer Complexes to DNA: Thermodynamic Base and Sequence Selectivity
Source: Int J Mol Sci. 2018 Jul 23;19(7):2137. doi: 10.3390/ijms19072137 (PMC6073332; doi:10.3390/ijms19072137)
Supplement: Supplementary file 1 [file ijms-19-02137-s001.pdf]

Supplementary materials

Binding of Organometallic Ruthenium Anticancer Complexes to DNA: A Thermodynamic Aspect on Base and Sequence Selectivity

Suyan Liu [1,3], Aihua Liang,[1] Kui Wu [2,3] *, Wenjuan Zeng [3, 4], Qun Luo [3, 4], Fuyi Wang [3, 4,] *

[1] Institute of Chinese Materia Medica, China Academy of Chinese Medical Sciences, Beijing 100700, PR China; syliu@icmm.ac.cn; ahliang@icmm.ac.cn

[2] School of Chemistry and Chemical Engineering, Wuhan University of Science and Technology, Wuhan 430081, PR China; wukui@wust.edu.cn

[3] Beijing National Laboratory for Molecular Sciences；National Centre for Mass Spectrometry in Beijing; CAS Key Laboratory of Analytical Chemistry for Living Biosystems, CAS Research/Education Centre for Excellence in Molecular Sciences, Institute of Chemistry, Chinese Academy of Sciences, Beijing 100190, PR China; zengwj2014@iccas.ac.cn; qunluo@iccas.ac.cn; fuyi.wang@iccas.ac.cn;

[4] University of Chinese Academy of Sciences, Beijing 100049, PR China.

* Correspondence: fuyi.wang@iccas.ac.cn; wukui@wust.edu.cn;

Tel.: +86-10-6252-9069

Table S1. Equilibrium binding constants for the reactions of complex **4** with different one-G-centered single-stranded ODNs (**I** and **IV**-**IX**). The general sequence of ODNs is 5′-CTCTCTXG_8YCTTCTC-3′.

ODN	K_1 (10^4 M^{-1})	K_2 (10^4 M^{-1})	K_1/K_2
-$C_7G_8A_9$- (**V**)	2.81 ± 0.55	2.59 ± 0.55	1.08
-$T_7G_8T_9$- (**I**)	2.92 ± 0.46	2.75 ± 0.47	1.06
-$A_7G_8C_9$- (**VI**)	3.33 ± 0.37	1.92 ± 0.24	1.73
-$A_7G_8A_9$- (**IX**)	3.61 ± 0.39	1.62 ± 0.20	2.22
-$T_7G_8A_9$- (**IV**)	3.99 ± 0.31	1.90 ± 0.16	2.10
-$C_7G_8C_9$- (**VII**)	4.75 ± 0.53	2.07 ± 0.25	2.29
-$A_7G_8T_9$- (**VIII**)	5.74 ± 0.44	2.20 ± 0.18	2.61

Table S2. Negatively-charged ions observed by HPLC-ESI-MS for SVP digests of ruthenated **II** produced by reaction of complex **1** or **2** with strand **II (**Ru/**II** = 3.0). The scheme below shows the representation of exonuclease digestion of single-stranded ODN **II** by SVP (**F$_i$** indicates the 5′-side fragment). **1′** = {(η^6-benzene)Ru(en)}$^{2+}$, **2′** = {(η^6-*p*-cymene)Ru(en)}$^{2+}$.

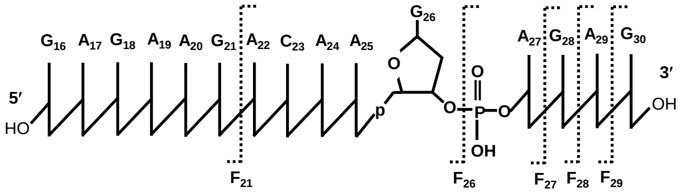

Ru complexes	Observed (Calculated) *m/z*	Ion Fragments
**1**	1050.53 (1050.18)	[F_{21} + **1′**]$^{2-}$
**2**	1078.24 (1078.21)	[F_{21} + **2′**]$^{2-}$
	1225.29 (1225.25)	[F_{21} + **2′**$_2$]$^{2-}$
	1335.63 (1335.59)	[F_{26} + **2′**$_2$]$^{3-}$

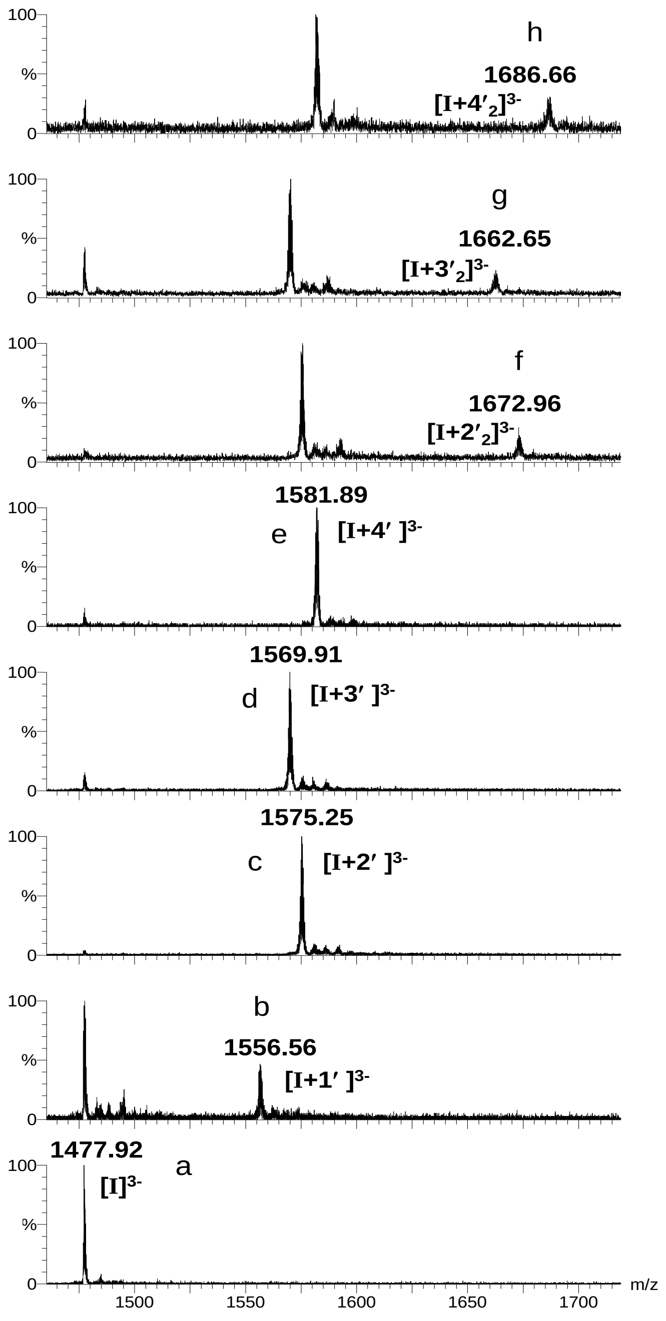

Figure S1. Mass spectra for HPLC fractions shown in Figure 1. **1′** = $\{(\eta^6\text{-benzene})Ru(en)\}^{2+}$, **2′** = $\{(\eta^6\text{-}p\text{-cymene})Ru(en)\}^{2+}$, **3′** = $\{(\eta^6\text{-indane})Ru(en)\}^{2+}$, **4′** = $\{(\eta^6\text{-biphenyl})Ru(en)\}^{2+}$.

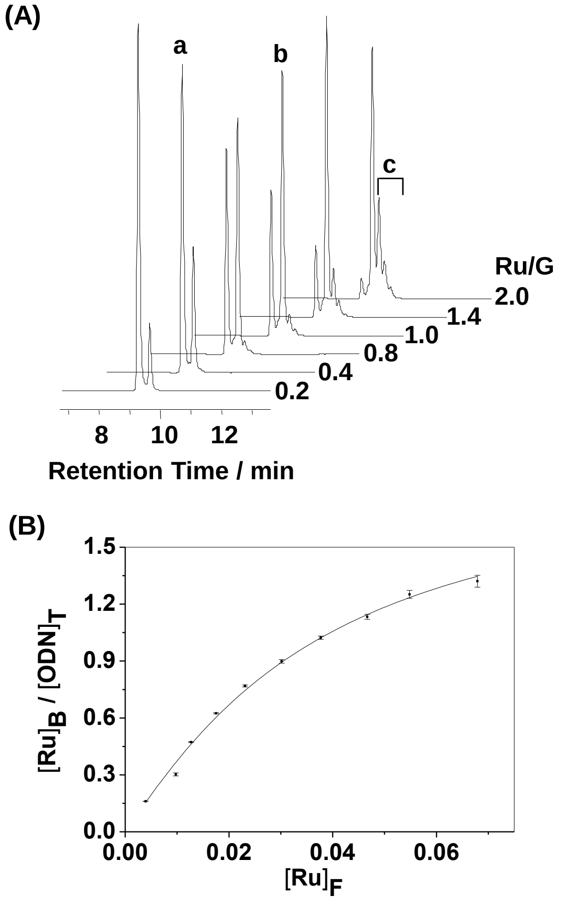

Figure S2. (A) HPLC chromatograms for reactions of complex **4** with single strand -TGA- (0.1 mM) at various molar ratios in 50 mM TEAA (pH 7) at 310 K for 24 h. Peak assignments: a, unruthenated -TGA-; b, mono-ruthenated -TGA-; c, di-ruthenated -TGA-; (B) Plot (dots) of DNA-bound **4** as a function of the concentration of free ruthenium complexes. Computer-fitting (line) of the experimental data to the ligand-receptor binding equation 8 gave rise to the equilibrium constants for the reaction of **4** with the ODN -TGA- listed in Table S3.

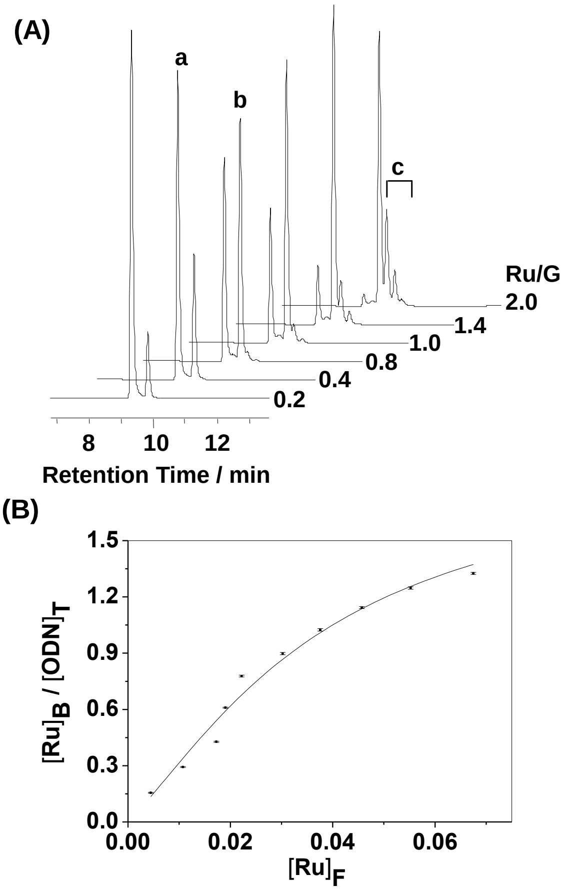

Figure S3. (A) HPLC chromatograms for reactions of complex **4** with single strand -CGA- (0.1 mM) at various molar ratios in 50 mM TEAA (pH 7) at 310 K for 24 h. Peak assignments: a, unruthenated -CGA-; b, mono-ruthenated -CGA-; c, di-ruthenated -CGA-; (B) Plot (dots) of DNA-bound **4** as a function of the concentration of free ruthenium complexes. Computer-fitting (line) of the experimental data to the ligand-receptor binding equation 8 gave rise to the equilibrium constants for the reaction of **4** with the ODN -CGA- listed in Table S3.

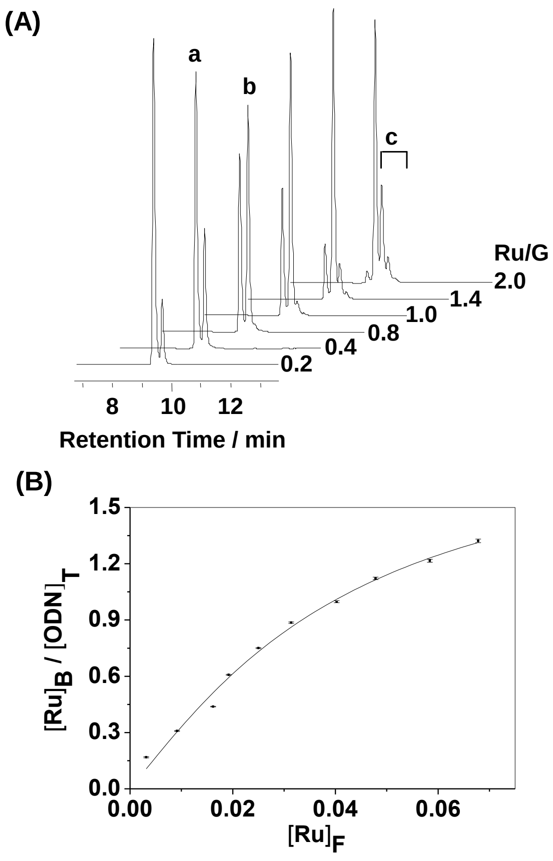

Figure S4. (A) HPLC chromatograms for reactions of complex **4** with single strand -AGC- (0.1 mM) at various molar ratios in 50 mM TEAA (pH 7) at 310 K for 24 h. Peak assignments: a, unruthenated -AGC-; b, mono-ruthenated -AGC-; c, di-ruthenated -AGC-; (B) Plot (dots) of DNA-bound **4** as a function of the concentration of free ruthenium complexes. Computer-fitting (line) of the experimental data to the ligand-receptor binding equation 8 gave rise to the equilibrium constants for the reaction of **4** with the ODN -AGC- listed in Table S3.

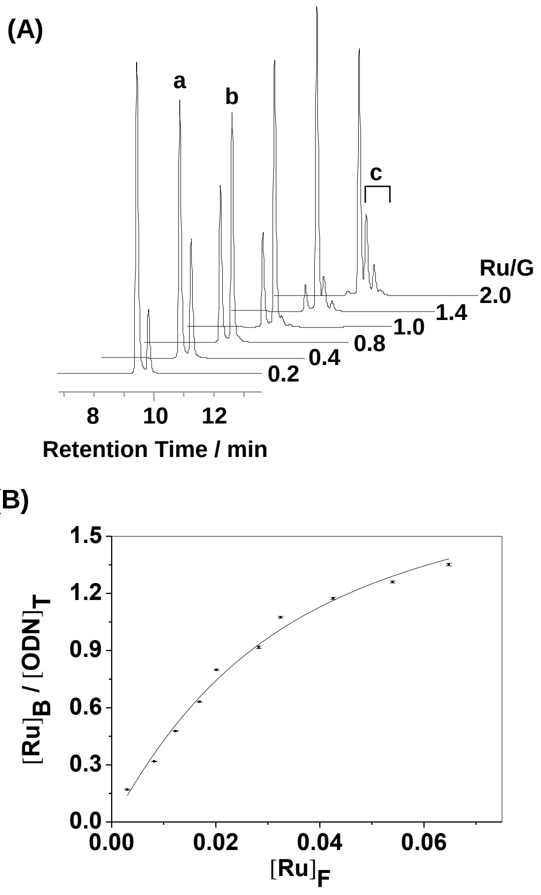

Figure S5. (A) HPLC chromatograms for reactions of complex **4** with single strand -CGC- (0.1 mM) at various molar ratios in 50 mM TEAA (pH 7) at 310 K for 24 h. Peak assignments: a, unruthenated -CGC-; b, mono-ruthenated -CGC-; c, di-ruthenated -CGC-; (B) Plot (dots) of DNA-bound **4** as a function of the concentration of free ruthenium complexes. Computer-fitting (line) of the experimental data to the ligand-receptor binding equation 8 gave rise to the equilibrium constants for the reaction of **4** with the ODN -CGC- listed in Table S3.

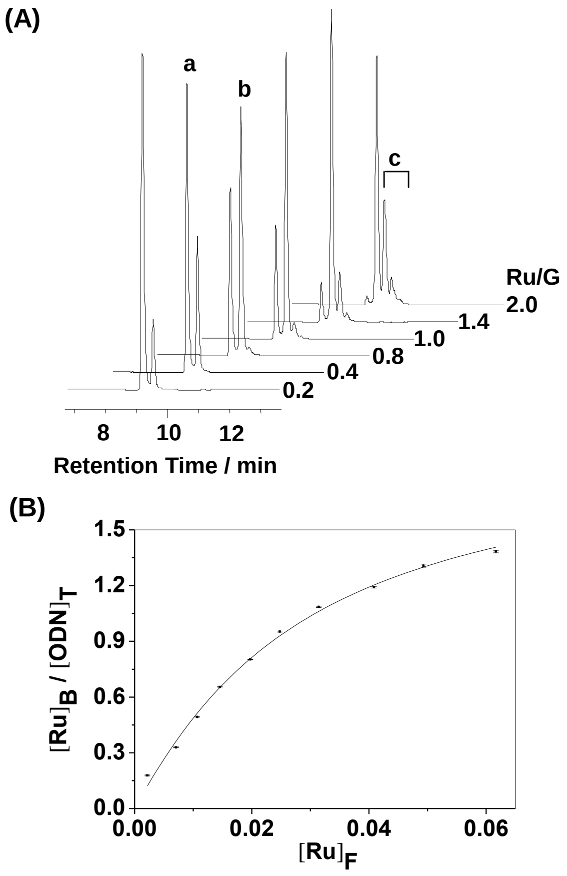

Figure S6. (A) HPLC chromatograms for reactions of complex **4** with single strand -AGT- (0.1 mM) at various molar ratios in 50 mM TEAA (pH 7) at 310 K for 24 h. Peak assignments: a, unruthenated -AGT-; b, mono-ruthenated -AGT-; c, di-ruthenated -AGT-; (B) Plot (dots) DNA-bound **4** as a function of the concentration of free ruthenium complexes. Computer-fitting (line) of the experimental data to the ligand-receptor binding equation 8 gave rise to the equilibrium constants for the reaction of **4** with the ODN -AGT- listed in Table S3.

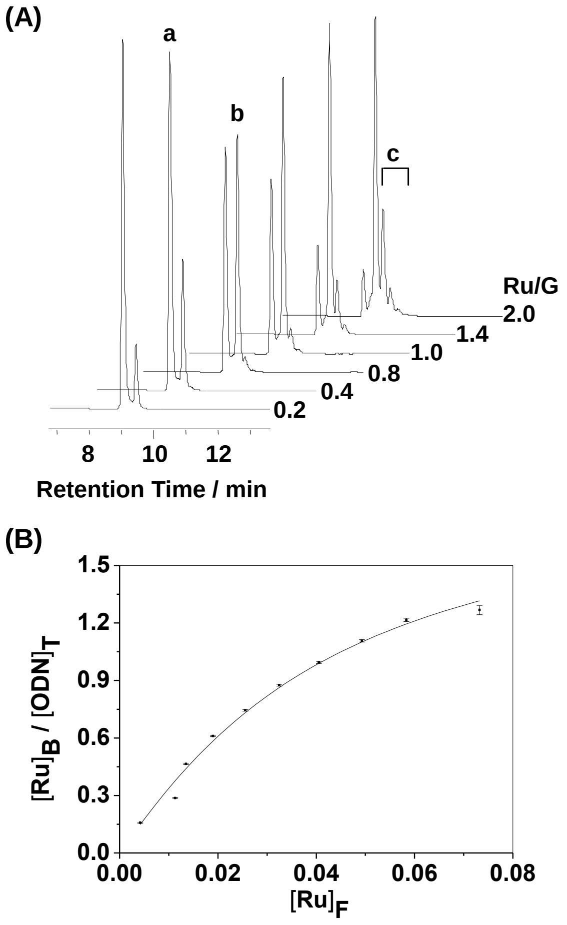

Figure S7. (A) HPLC chromatograms for reactions of complex **4** with single strand -AGA- (0.1 mM) at various molar ratios in 50 mM TEAA (pH 7) at 310 K for 24 h. Peak assignments: a, unruthenated -AGA-; b, mono-ruthenated -AGA-; c, di-ruthenated -AGA-; (B) Plot (dots) DNA-bound **4** as a function of the concentration of free ruthenium complexes. Computer-fitting (line) of the experimental data to the ligand-receptor binding equation 8 gave rise to the equilibrium constants for the reaction of **4** with the ODN -AGA- listed in Table S3.

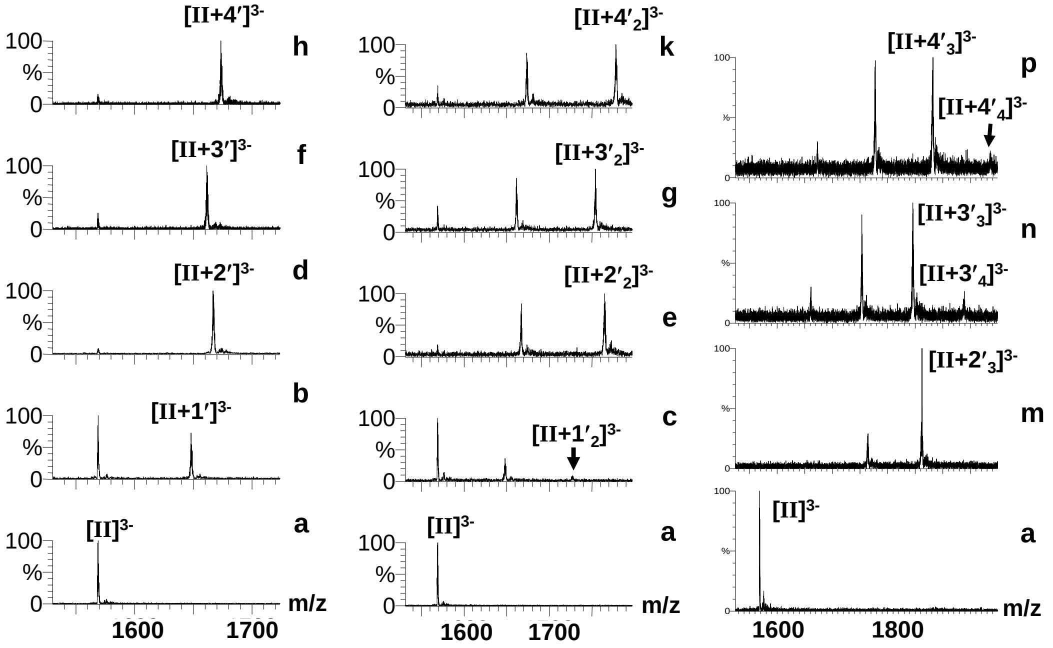

Figure S8. Mass spectra for HPLC fractions showed in Figure 5. **1′** = {(η^6-benzene)Ru(en)}$^{2+}$, **2′** = {(η^6-*p*-cymene)Ru(en)}$^{2+}$, **3′** = {(η^6-indane)Ru(en)}$^{2+}$, **4′** = {(η^6-biphenyl)Ru(en)}$^{2+}$.

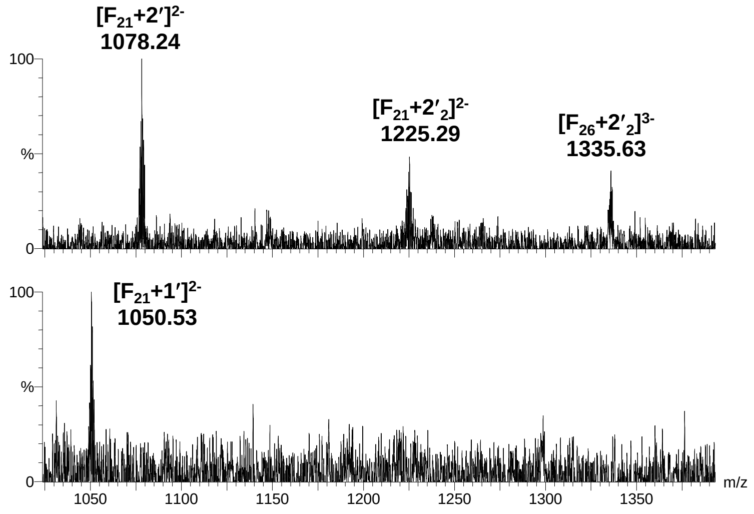

Figure S9. Mass spectra for ruthenated oligonucleotide fragments F_{21} and F_{26} arising from SVP digestion of ruthenated **II** by complex **1** or **2**. **1′** = {(η^6-benzene)Ru(en)}$^{2+}$, **2′** = {(η^6-*p*-cymene)Ru(en)}$^{2+}$.

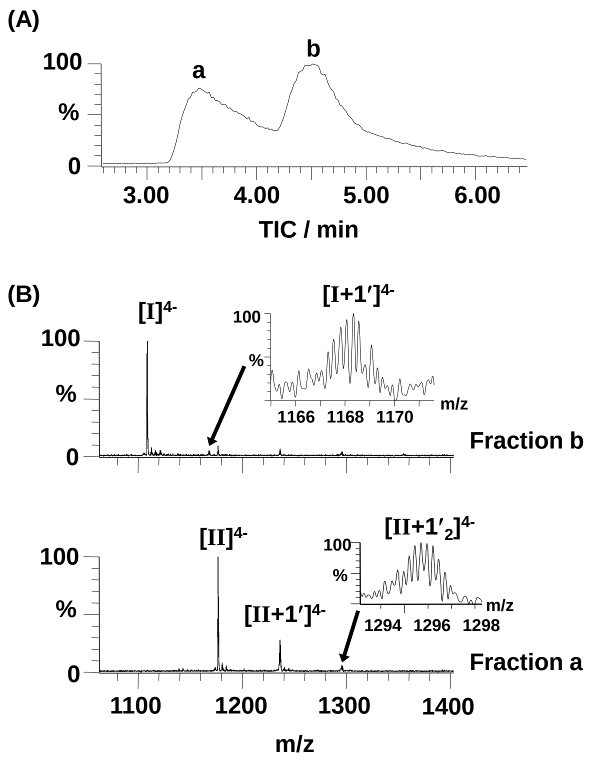

Figure S10. (A) Chromatograms with total ion count (TIC) detection for the reaction mixture of complex **1** and 15-mer duplex **III** (Ru/**III** = 6.0) in 50 mM TEAA buffer (pH 7) and 100 mM $NaClO_4$ incubated at 310 K for 48 h. (B) Mass spectra for HPLC fractions shown in (A).

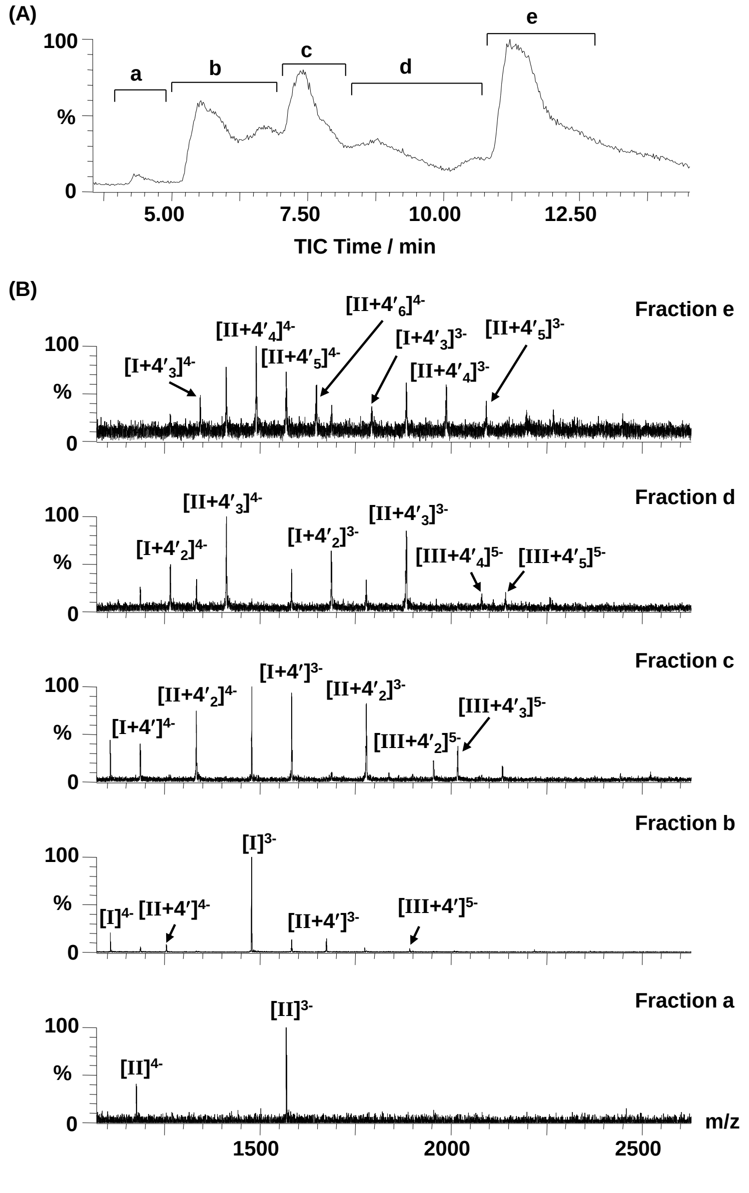

Figure S11. (A) Chromatogram with total ion count (TIC) detection for the reaction mixture of complex **4** and duplex **III** (Ru/**III** = 6.0) in 50 mM TEAA buffer (pH 7) and 100 mM $NaClO_4$ incubated at 310 K for 48 h. (B) Corresponding mass spectra for HPLC fractions shown in (A).

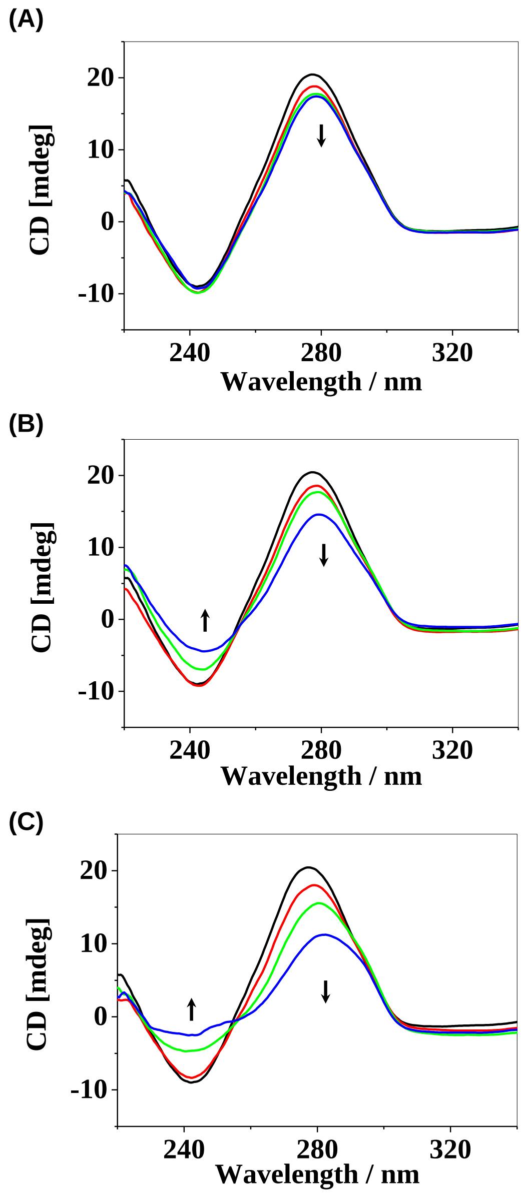

Figure S12. CD spectra of free duplex **III** (black) and the reaction mixtures of duplex **III** with complex **1** (A), **2** (B) or **4** (C) incubated under 310 K for 24 h at a molar ratio of Ru/**III** = 1.0 (red), 3.0 (green) and 6.0 (blue).
